# Perception of the oral health risks of passive smoking from traditional cigarettes, electronic cigarettes, and heated tobacco products: A cross-sectional study

**DOI:** 10.18332/tid/186588

**Published:** 2024-05-02

**Authors:** Francesco Saverio Ludovichetti, Andrea Zuccon, Adolfo Di Fiore, Giulia Zambon, Adriana Bargan, Edoardo Stellini, Sergio Mazzoleni

**Affiliations:** 1 Section of Dentistry, Department of Neurosciences, University of Padova, Padova, Italy

**Keywords:** passive smoking, oral health, IQOS, traditional cigarettes, electronic cigarettes

## Abstract

**INTRODUCTION:**

Tobacco smoke is a major health risk factor for smokers but also for non-smokers due to passive smoking. These risks come from conventional cigarette smoke but also from aerosol produced by electronic cigarettes and heated tobacco products (HTPs). The aim of this study was to investigate population knowledge about the adverse effects of passive smoking from traditional cigarettes, electronic cigarettes, and HTPs.

**METHODS:**

Between February and October 2023, 504 subjects among the general population responded to a questionnaire with 8 questions in Italian, via a link to the Google Forms platform. The questions related to the oral health effects of active and passive smoking. Descriptive analyses of all variables in the questionnaire were performed, and statistical analyses between variables were carried out using the chi-squared test and Fisher's exact test.

**RESULTS:**

A large subset of individuals interviewed stated that active smoking is harmful to health and consider active smoking more damaging compared with passive smoking (86.3%). The majority believed that passive smoking of cigarettes is more harmful to oral health than passive smoking of HTPs (79.4%) or electronic cigarettes (e-cigarettes) (84.9%).

**CONCLUSIONS:**

Results suggest that most people in this study had good knowledge about the adverse effects of active or passive smoking on health; however, knowledge regarding e-cigarettes and HTPs was poor and confused. These results reveal the complexity of perceptions regarding different types of smoking and the need for further research to fully understand the risks associated with each type of passive smoking.

## INTRODUCTION

Globally, tobacco smoking is one of the most significant public health problems, increasing the risk of several oral and systemic diseases. Tobacco smoke is a risk factor for smokers but also for non-smokers, with an increased risk of health problems due to involuntary exposure to passive tobacco smoking. Passive tobacco smoke mainly occurs from the smoking part of cigarettes between puffs (sidestream smoke, SS) and smoke exhaled by smokers (mainstream smoke, MS). Passive smoke is composed of 15% MS and 85% SS^1^. Children are more vulnerable to the dangerous effects of passive smoking. Approximately 40% of children are exposed to passive smoking globally, with particularly high percentages found in Europe (77.8%) and the Western Pacific (50.6%)^2^. Children exposed to passive smoking show a greater incidence of dental caries, with a significantly higher Decay, Missing, Filled Teeth (DMFT) index compared to control groups not exposed to passive smoking^3,4^. The dose-response relationship between the level of passive smoking exposure and DMFT scores provides further supporting evidence. Also, the increase in caries in teenagers has been hypothesized to be associated with such exposure^5^. Additionally, passive smoking has been associated with multiple oral diseases, including halitosis, reduced taste perception, teeth staining, leukoplakia, and oral carcinoma^6^.

Electronic cigarettes, similar to traditional cigarettes, produce an aerosol containing significant levels of harmful chemicals^7^. Increased nicotine levels in saliva and urine have been observed in non-smokers exposed to e-cigarette emissions^8^. In recent times, HTPs, often referred to as a modified-risk product (MRP), have been introduced. These products heat tobacco to 350°C to reduce toxic substances compared to conventional cigarettes^9^.

This study aims to explore how a sample of Italian adults categorized the oral health effects of passive smoking of traditional cigarettes, electronic cigarettes, and HTPs.

## METHODS

In this cross-sectional study, a questionnaire was created and administered to a convenience sample of 504 participants. The questionnaire was administered in Italian via a link to the Google Forms platform. Data collection was from February to October 2023. The questionnaire was open to the general population, anonymous, and all participants were guaranteed complete confidentiality of the information.

One answer was required for each proposed question; therefore, partially submitting the questionnaire was not possible. Two sections of the questionnaire relate to active smoking and passive smoking. It consisted of 8 questions, 2 of which were multiple-choice and six single-choice. A score from 1 to 7 was created based on the number of items the respondents reported correctly to the question: ‘Which of these negative effects of active smoking do you know?’. Responses included oral carcinoma, periodontal disease, caries, teeth pigmentation, smoke-related melanosis, nicotinic stomatitis, and asthma. Subjects were categorized according to the level of knowledge they had about the adverse effects of smoking: 1 correct response ‘very low knowledge’, 2–3 ‘low knowledge’, 4–5 ‘reasonable’, and 6–7 ‘high level of knowledge’ (Supplementary file Figure 1).

### Statistical analysis

Statistical tests used in this study were performed through the statistical program R (Version 4.3.2). Descriptive analyses of all variables in the questionnaire were performed, and statistical analyses between variables were carried out by chi-square test and Fisher’s exact test with a level of statistical significance α=0.05.

## RESULTS

Descriptive analyses of responses to the eight questions in the questionnaire are provided in Supplementary file Part A. Nearly all respondents (99.6%) acknowledged the detrimental impact of smoking on health, with 86.3% asserting that active smoking is more harmful than passive smoking. A substantial portion (79.4%) believed that passive smoking from conventional cigarettes poses a greater risk to oral health than exposure to HTPs. In comparison, 84.9% considered it more harmful than passive smoking from electronic cigarettes, while 71.8% did not perceive passive smoking from e-cigarettes as more dangerous than that from HTPs.

Supplementary file Part B displays the correlation between variables. Out of 504 respondents, only 2 asserted that passive smoking from regular cigarettes is not dangerous. At the same time, the majority (n=399) believed it to be more harmful than passive smoking from HTPs, while 28.2% indicated that passive smoking from electronic cigarettes is more harmful to oral health than passive smoke from HTPs.

The participants were categorized based on their knowledge of the adverse effects of smoking. Of the 504 individuals, 36.5% were classified as having reasonable knowledge. Among these, 150 agreed that passive smoking from regular cigarettes is more harmful than from e-cigarettes for oral health. A statistically significant association (p=0.024) was found between knowledge level and the belief that passive smoking from regular cigarettes is more harmful than from e-cigarettes (Supplementary file Part A). In the ‘very low knowledge’ group, 26 out of 30 believed passive smoking from traditional cigarettes is more harmful than HTPs. However, no significant association (p=0.387) was observed between the level of knowledge and opinions on the harm of passive smoking from traditional cigarettes compared to HTPs.

The ‘reasonable knowledge’ group had the most disagreements between the level of knowledge and opinions on the harm of passive smoking, while the ‘high knowledge’ and ‘low knowledge’ groups had individuals agreeing, but the association was not statistically significant (p=0.072).

Only 13.7% of the convenience sample believed passive smoking to be more harmful than active smoking. While 315 respondents agree that active smoking is more harmful than passive smoking, no statistically significant difference was found among the analyzed variables (p>0.05).

## DISCUSSION

Our study indicates that most people in our sample of Italian adults perceived active smoking as hazardous, with the majority considering passive smoke from regular cigarettes more harmful to oral health than HTPs. In alignment with our findings, Park et al.^10^ observed a more positive perception of HTPs, particularly among users who rated them favorably compared to traditional cigarettes. HTP users report fewer odors, smoke, and passive smoke compared to cigarettes. Evidence indicates lower exposure to toxic substances in HTPs than in conventional cigarettes^11^. However, it is important to note that a considerable part of this evidence comes from the tobacco industry. Considerable questions about their reliability are consequently raised^12^. Limited research explores HTPs’ perceived harm versus traditional cigarettes and e-cigarettes. Sutanto et al.^12^ report that most consider e-cigarettes and HTPs equally harmful but perceive e-cigarettes as less harmful than traditional cigarettes^12^. Similar results were also found in a study by Jankowsi et al.^13^, in which it was observed that e-cigarettes and HTPs were perceived to be less harmful than traditional cigarettes. Pollution levels from HTPs and e-cigarettes have been previously investigated and it was observed that smoking e-cigarettes inside vehicles led to a significant increase in the concentration of PM2.5 particles and >300 nm particles. In contrast, HTP aerosol was measured to have almost no impact on the concentration of fine particles (>300 nm) or the concentration of PM2.5^14^. Electronic cigarettes emit an aerosol that includes fine and ultrafine liquid particles from over-saturated propylene glycol. Prolonged exposure to these particles in children and adolescents could be potentially harmful and increase the risk of asthma^15^. In addition, e-cigarettes release volatile organic compounds (VOCs), which may reach amounts of 795.4 mg/100 puffs, about 9.5 times higher than those released by traditional cigarettes^16^.

In our study, those well-versed in the adverse effects from active smoking deemed smoke from regular cigarettes more harmful to the oral cavity than that from e-cigarettes. However, the present study did not find a significant association between the level of knowledge about the adverse effects of active smoking and opinions that passive smoking from traditional cigarettes is more harmful buccally than passive smoking from HTPs. In a survey conducted by Istenic et al.^17^, it was found that most respondents had good knowledge about the adverse health effects of smoking. In our study, 76.6% recognized oral cancer risks, 91.7% identified harm during pregnancy, and 93% the link between smoking and teeth issues. Limited awareness exists about passive smoking and product distinctions. Another study found that 20.4% deemed HTPs safe for passive smokers^18^. HTP users perceive these products as less harmful, aligning with industry promotion as a safer alternative. Despite FDA-identified harmful components, an independent study found higher levels of 53 substances in HTP aerosol than in regular cigarettes in 2018^19^.

### Strengths and limitations

This cross-sectional study expeditiously captures public perceptions and knowledge regarding health risks associated with smoking, e-cigarettes, and HTP use on oral health. However, the utilization of self-reported data introduces potential response bias, recall bias and social desirability biases, while the non-random sampling design applied limits the generalizability of the results. Constrained by a focus on an Italian-speaking population, the study’s geographical scope may limit the study’s broader applicability. Additionally, the sample size may inadequately represent the overall population diversity.

## CONCLUSIONS

Tobacco smoke poses significant health risks for both smokers and non-smokers through passive exposure. This study assessed public awareness of oral health effects from passive smoking of traditional cigarettes, electronic cigarettes, and HTPs. Most participants perceived active smoking as more harmful than passive smoking, with traditional cigarette smoke seen as more harmful than exposure to electronic cigarettes and HTPs. While respondents were well aware of active and passive smoking risks, knowledge regarding e-cigarettes and HTPs was limited. These findings underscore varied perceptions across smoking forms, emphasizing the need for further research to understand associated risks comprehensively.

## Supplementary Material



## Data Availability

The data supporting this research can be found in the Supplementary file.
