# Supplementary material for: Perception of the oral health risks of passive smoking from traditional cigarettes, electronic cigarettes, and heated tobacco products: A cross-sectional study
Source: Tob Induc Dis. 2024 May 2;22:10.18332/tid/186588. doi: 10.18332/tid/186588 (PMC11064127; doi:10.18332/tid/186588)
Supplement: Supplementary file 1 [file TID-22-71-s1.pdf]

Supplementary Figure 1.

Figure 1. Question 7: "Which of these negative effects of active smoking do you know?", multiple choice question with 7 options (A). Based on the number of items known, a score from 1 to 7 was created. Subjects were ranked according to the level of knowledge they had about negative effects of smoking (B).

A)

**Options**
Oral carcinoma
Periodontal disease
Caries
Teeth pigmentation
Smoke-related melanosis
Nicotinic stomatitis
Asthma

B)

**Number of chosen options**	**Level of knowledge**
1	Very low
2–3	Low
4–5	Reasonable
6–7	High

Part A: Questions

1. Question 1

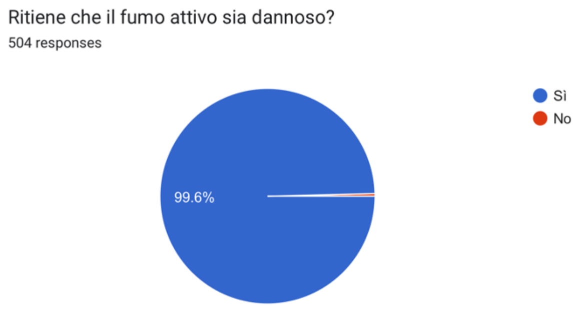

2. Question 2

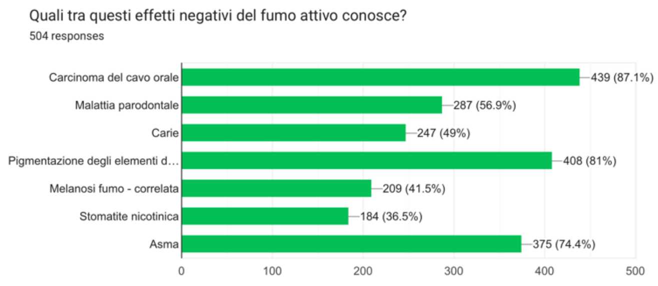

3. Question 3

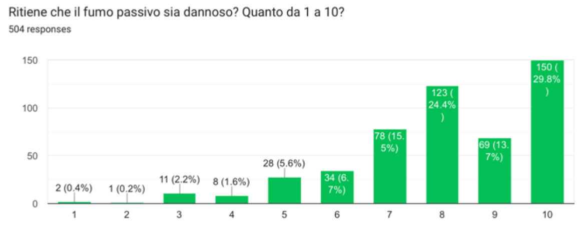

4. Question 4

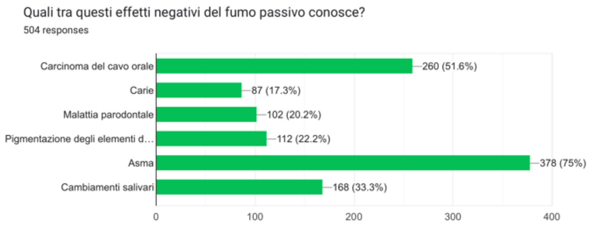

5. Question 5

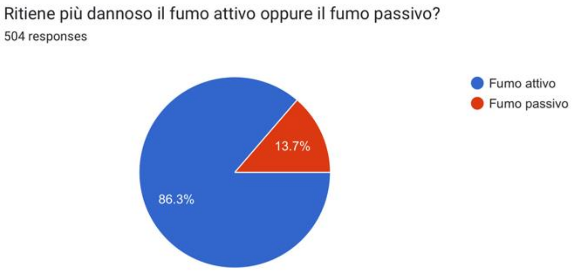

6. Question 6

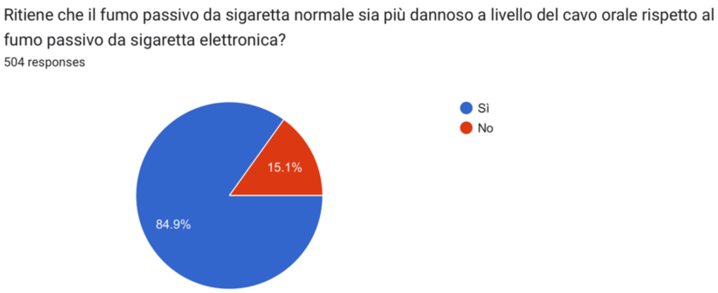

7. Question 7

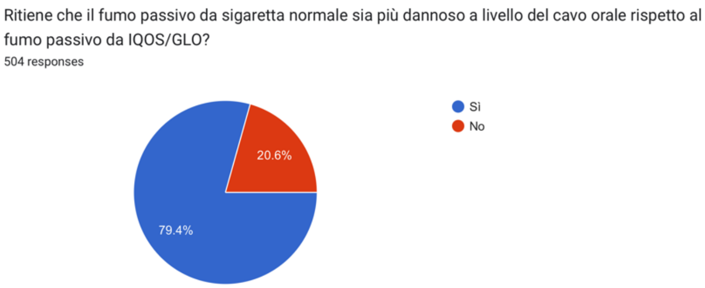

8. Question 8

Ritiene che il fumo passivo da sigaretta elettronica sia più dannoso a livello del cavo orale rispetto al fumo passivo da IQOS/GLO?

504 responses

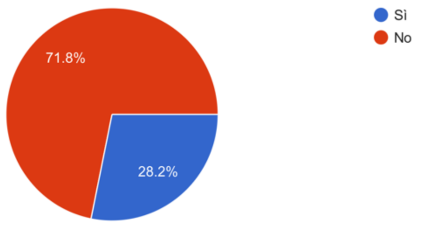

Part B: Contingency tables between the categorical variables

Do you think active smoking is harmful?"	Do you think passive smoking from regular cigarettes is more harmful to the oral cavity than passive smoking from IQOS/GLO?		Total
	No	Yes	
NO	1	1	**2**
YES	103	399	**502**
**Total**	**104**	**400**	**504**
**Fisher Test - p-value = 0.3704**			

Do you think active smoking is harmful?	Do you think passive smoking from e-cigarettes is more harmful in the mouth than passive smoking from IQOS/GLOs?		Total
	No	Yes	
NO	2	0	**2**
YES	360	142	**502**
**Total**	**362**	**142**	**504**

Fisher Test - p-value = 0.98

Level of knowledge about how active smoking negatively impacts on us	Do you feel that passive smoking from regular cigarettes is more harmful to the oral cavity than passive smoking from electronic cigarettes?		Total
	No	Yes	
Very low	4	26	**30**
Low	12	137	**149**
Reasonable	34	150	**184**
High	26	115	**141**
**Total**	**76**	**428**	**504**
**Chisq Test - p-value = 0.024**			

Level of knowledge about how active smoking negatively impacts on us	Do you consider passive smoking from regular cigarettes to be more harmful in the oral cavity than passive smoking from IQOS/GLOs?		Total
	No	Yes	
Very low	4	26	**30**
Low	26	123	**149**
Reasonable	40	144	**184**
High	34	107	**141**
**Total**	**104**	**400**	**504**
**Chisq Test - p-value =**	**0.387**		

Level of knowledge about how active smoking negatively impacts on us	Do you feel that passive smoking from e-cigarettes is more harmful to oral cavity than passive smoking from IQOS/GLOs?		Total
	No	Yes	
Very low	25	5	**30**
Low	96	53	**149**
Reasonable	135	49	**184**
High	106	35	**141**
**Total**	**362**	**142**	**504**
**Chisq Test - p-value = 0.072**			

Do you consider active smoking or passive smoking to be more harmful?	Do you think passive smoking from regular cigarettes is more harmful to oral cavity than passive smoking from electronic cigarettes?		Total
	No	Yes	
Active	65	370	**435**
Passive	11	58	**69**
**Total**	**76**	**428**	**504**
**Chisq Test - p-value = 0.973**			

Do you consider active smoking or passive smoking to be more harmful?	Do you think passive smoking from regular cigarettes is more harmful to oral cavity than passive smoking from IQOS/GLO?	Total

	No	Yes	
Active	87	348	**435**
Passive	17	52	**69**
**Total**	**104**	**400**	**504**
**Chisq Test - p-value = 0.423**			

**Do you consider active smoking or passive smoking to be more harmful?**	**Do you think passive smoking from electronic cigarettes is more harmful to oral cavity than passive smoking from IQOS/GLO?**		**Total**
	No	Yes	
Active	315	120	**435**
Passive	47	22	**69**
**Total**	**362**	**142**	**504**
**Chisq Test - p-value = 0.553**			

© **2024 Ludovichetti F. S. et al.**
